# Deep learning-enhanced image registration for accelerating daily adaptive magnetic resonance imaging-guided prostate radiotherapy

**DOI:** 10.1016/j.phro.2026.101052

**Published:** 2026-08-02

**Authors:** Cornel Zachiu, Gijsbert H. Bol, Alexis N.T.J. Kotte, Thomas Willigenburg, Matteo Maspero, Mark H.F. Savenije, Johannes C.J. de Boer, Jochem R.N. van der Voort van Zyp, Cornelis A.T. van den Berg, Bas W. Raaymakers

**Affiliations:** Department of Radiotherapy, UMC Utrecht, Heidelberglaan 100, 3508 GA, The Netherlands

**Keywords:** Daily auto-contouring, Prostate radiotherapy, MR guidance, Online adaption

## Abstract

**Background and purpose:**

Daily auto-contouring remains a workflow bottleneck in magnetic resonance-guided adaptive prostate radiotherapy (MRgRT). This study proposes and clinically validates a novel deep learning-enhanced deformable image registration (DIR) solution to accelerate this critical step.

**Materials and Methods:**

A hybrid framework combining a 3D nnU-Net segmenting bladder/rectum on planning/daily MRI with an in-house DIR algorithm was implemented for 5-fraction prostate MRgRT (5×7.25 Gy) on an MR-Linac. The DIR uses nnU-Net contours to propagate target and organs-of-interest structures. The solution was clinically deployed and evaluated in 275 patients/1375 fractions.

**Results:**

Evaluation following clinical introduction, has shown a median contouring time of ≈190 s, halving the time required by the previously-employed vendor-provided solution. Quantitative evaluation showed high agreement with clinically approved contours: Dice similarity coefficients >0.9 and 95th percentile Hausdorff distances <2.0 mm for most structures.

**Conclusions:**

The implemented solution demonstrated reliable, high-accuracy daily auto-contouring, significantly accelerating MRgRT workflows. It has become our institutional standard for prostate treatments. Future work will extend this approach to additional treatment sites and modalities.

## Highlights

• Daily contouring is a bottleneck for MRI-guided prostate radiotherapy.
• A new deep learning-enhanced contour propagation solution is proposed.
• The software was clinically-implemented and used in the treatment of 275 patients.
• The median contouring time was reduced from 7.8 min to 3.1 min.

## Graphical abstract

**Figure:**
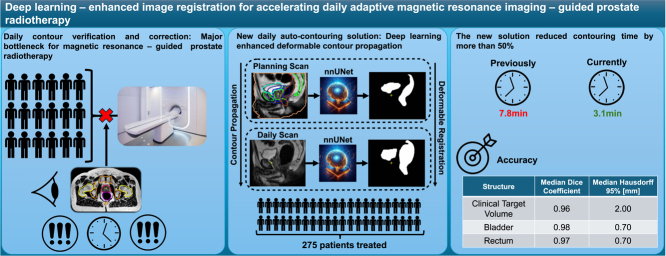

## 1 Introduction

The clinical implementation of magnetic resonance (MR)-guided radiotherapy (RT) for prostate cancer has enabled daily adaptation of treatment plans based on the patient’s anatomy at the time of delivery [1], [2]. This process typically involves the acquisition of a 3D MR image at the start of each treatment fraction, allowing clinicians to account for positional and anatomical variations relative to the initial offline plan [3], [4]. Based on the new image, relevant structures are identified and, if necessary, re-contoured, followed by the adaptation or re-optimization of the treatment plan. Accurate delineation of target areas and organs-of-interest (OoI) on the daily anatomy is therefore a critical component of the adaptive workflow.

While manual contouring remains the standard, it is a logistical burden for online adaptive MR-guided RT due to its time-intensive nature. Consequently, most clinical systems incorporate automatic delineation tools to facilitate contouring [5], [6]. Historically, many of these tools have approached the problem as one of deformable image registration (DIR) [7], [8], [9], [10], wherein deformation fields are estimated between a reference image — typically a pre-treatment planning MR or an atlas — and the daily image. These deformations are then applied to propagate the reference contours onto the anatomy-of-the-day. Although registration-based methods have shown utility, recent advances have favored deep learning (DL)-based approaches that recast daily contouring as a direct image-to-contour problem [5], [11], [12], [13]. In addition to their superior computational efficiency, DL methods have demonstrated improved accuracy, particularly in regions with complex deformations where DIR techniques often struggle [11], [14]. However, DL models typically require retraining in response to changes in imaging protocols, which can be challenging due to limited availability of annotated training data. Moreover, in the context of MR-guided prostate RT, DL methods tend to perform well in segmenting OoI but face challenges in reliably identifying the gross tumor volume (GTV) and other structures with less distinct anatomical boundaries, such as the clinical target volume (CTV) [11], [13], [15]. In such cases, DIR-based methods offer an advantage by leveraging the accurate planning contours, which can be propagated onto daily anatomy.

Regardless of the approach however, auto-generated contours from currently available vendor-provided solutions often require manual adjustments prior to clinical approval. These adjustments can introduce several minutes of delay per fraction, being identified as a significant bottleneck in daily-adaptive workflows [9], [12], [13], [14].

The aim of this study was hereby to propose a fast and accurate daily auto-contouring framework that integrates the complementary advantages of deep learning and deformable image registration. Specifically, a DL network was first used to segment the OoI, and the resulting contours were used as guidance for an in-house developed DIR algorithm. The framework was designed for high parallelizability and implemented on graphical processing units (GPUs), lending it suitable for online clinical use. Additionally, the performance of the framework was evaluated, both in terms of accuracy and contour verification time, following its clinical deployment within our department.

**Fig. 1:**
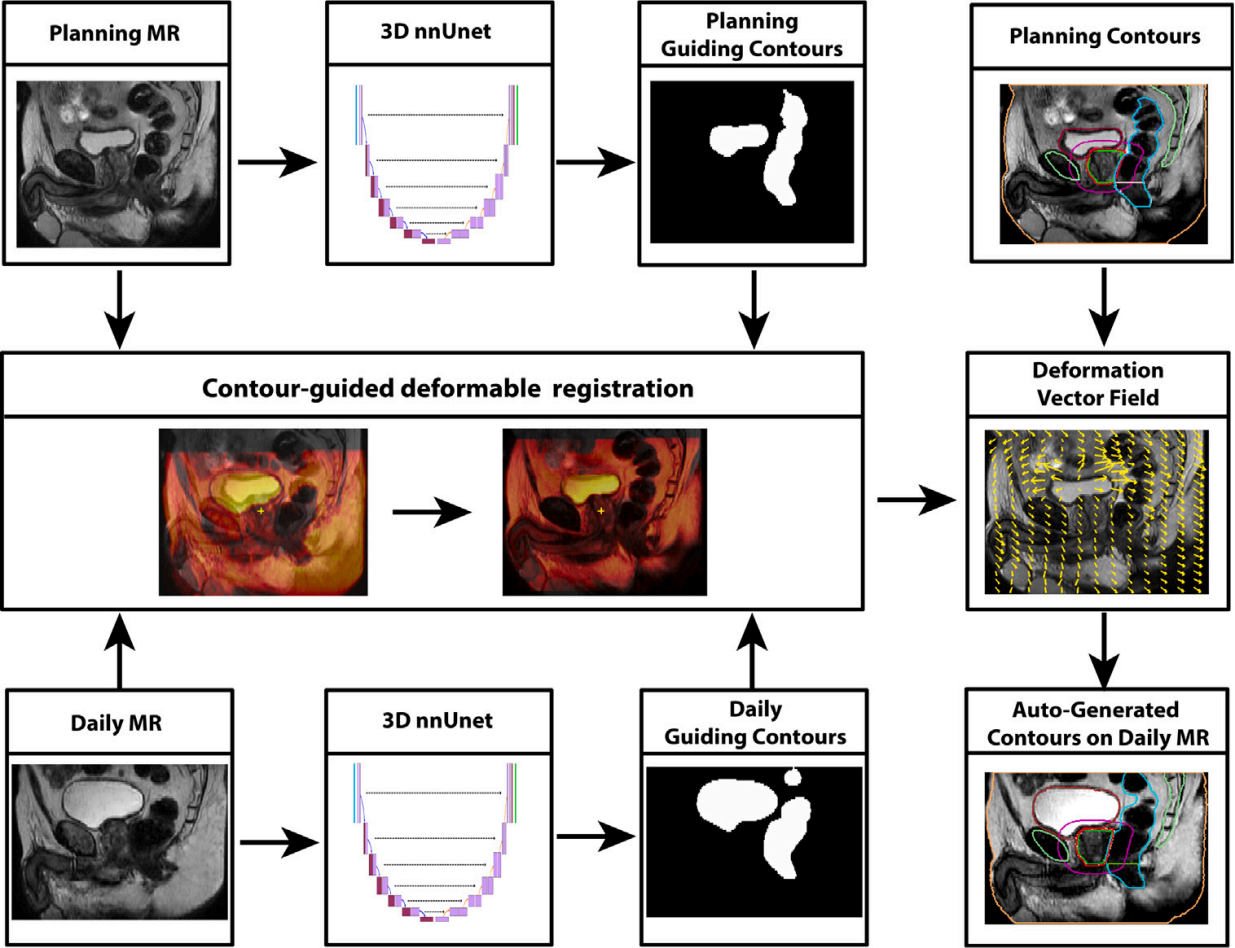
Summary of the EvoDL auto-contouring framework. An estimation of the bladder and rectum contours is generated by a 3D nnUnet on both the planning and the daily MR images. These are then used as guiding information for a deformable image registration algorithm. The resulting deformation vector fields are then used to propagate the planning contours onto the daily anatomy.

## 2 Materials & methods

### 2.1 Patient and imaging data

The population for which the proposed contouring framework was employed consisted of 275 intermediate-risk prostate cancer patients undergoing a 5×7.25 Gy treatment scheme on Elekta Unity MR linacs. Patient consent was obtained under the MOMENTUM study (NCT04075305) [16]. Specific details on the overall adaptive workflow and image acquisition protocol can be found in one of our previous studies [14]. In summary, at the start of each fraction, a 3D T2-weighted MR image was acquired, which was subsequently registered to a pre-treatment MR scan. The resulting estimated deformations were then used to propagate the pre-treatment contours onto the anatomy of the day, followed by verification, potential editing and approval by a radiation therapist (RTT). The acquisition parameters for the pre-treatment and daily MRI scans are provided in Table 1.

**Table 1:** MR acquisition parameters for the pre-treatment and daily MRI scans.

	Pre-treatment	Daily
Field strength [T]	3	1.5
Repetition time [ms]	1700	1450
Echo time [ms]	280	120
Flip angle [°]	90	90
Slice thickness [mm]	2	2
Reconstructed resolution [mm${}^{3}$]	0.57 × 0.57 × 2	0.69 × 0.69 × 2

### 2.2 Contour propagation

The proposed auto-contouring framework, named “EvoDL”, was designed as a hybrid between a neural network and an in-house developed deformable registration algorithm. A deep learning approach initially segmented the bladder and rectum contours on the planning and daily images [17]. The generated contours are used as constraints during the deformable registration methods adopted to perform contour propagation of all the contours available in the planning image (Fig. 1).

**Fig. 2:**
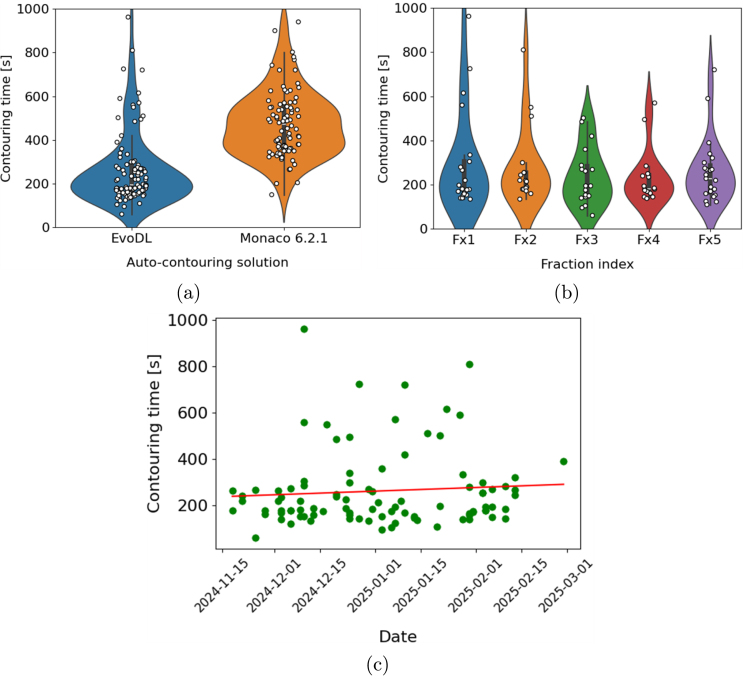
The time in seconds required for the contouring step of the adaptive MR-guided prostate RT workflow. (a) A direct comparison between EvoDL and Monaco 6.2.1. (b) The recorded time per fraction for EvoDL. (c) Long-term analysis of the contouring time over the first three and a half months following its clinical introduction.

#### 2.2.1 Auto-segmentation of the guiding contours

The segmentation of the bladder and rectum, used as constraints for the registration component of the proposed framework, was achieved by the means of a full resolution 3D nnUNet. The network was trained using pre-treatment T2-weighted MR images (see Table 1) and the corresponding clinical delineations performed by the treating radiation oncologist. The data originated from a total of 115 prostate cancer patients, treated prior to the clinical implementation of the proposed framework.

#### 2.2.2 Deformable image registration

A contour-guided gradient-based DIR algorithm, called Evolution [18], was used for registration. From a mathematical point-of-view, the algorithm estimates the set of deformations u as the minimizer of the following cost function:

(1) $$E\left(u\right)=\underset{\vec{r}\in \Omega }{\sum }\left(D\left[u\left(\vec{r}\right)\right]+\beta K\left[u\left(\vec{r}\right)\right]+\alpha {\left\|\nabla u\left(\vec{r}\right)\right\|}_{2}^{2}\right)$$

where

(2) $$D\left[u\left(\vec{r}\right)\right]=\exp\left\{-\frac{\underset{\vec{\gamma }\in \Gamma }{\sum }\left|\nabla I\left(\vec{r}+\vec{\gamma }\right)\cdot \nabla J\left(\vec{r}+\vec{\gamma }+u\left(\vec{r}+\vec{\gamma }\right)\right)\right|}{\left(\underset{\vec{\gamma }\in \Gamma }{\sum }{\left\|\nabla I\left(\vec{r}+\vec{\gamma }\right)\right\|}_{2}\right)\left(\underset{\vec{\gamma }\in \Gamma }{\sum }{\left\|\nabla J\left(\vec{r}+\vec{\gamma }+u\left(\vec{r}+\vec{\gamma }\right)\right)\right\|}_{2}\right)}\right\}$$

and

(3) $$K\left[u\left(\vec{r}\right)\right]={\left\{C_{I}\left(\vec{r}\right)-C_{J}\left(\vec{r}+u\left(\vec{r}\right)\right)\right\}}^{2}$$

The rest of the terms are as follows: α and β are weighting parameters, I and J are the planning and the daily T2-weighted MR images, Ω is the image domain, ∇ is the gradient operator, $\vec{r}$ and $\vec{\gamma }$ are position vectors, Γ is a neighborhood around the voxel at location $\vec{r}$, |⋅| is the absolute value function and ${\left\|\cdot \right\|}_{2}$ is the Euclidean norm. The term D[u] quantifies the mis-alignment between the two images, guiding the algorithm towards a set of deformations u which aligns similar contrast patterns in the two images, such as organ boundaries and high-contrast features.

The terms $C_{I}$ and $C_{J}$ are, what we will call in the scope of this manuscript, guiding contours and are defined on each of the two images being registered. The term K[u] therefore provides additional information on the probable location of a corresponding subset of anatomical structures in the two images and penalizes mis-alignments between them. In this study, the term K was defined based on the bladder and rectum contours, generated as described in Section 2.2.1.

#### 2.2.3 Numerical implementation

The minimization of the cost function from Eq. (1) was performed using the numerical scheme described in [18]. In summary, the optimal deformation vector fields (DVFs) u were determined by the means of a Gauss–Newton iterative solver, employing a coarse-to-fine numerical scheme with iterative refinement of the deformations at each resolution level [19], [20]. To balance computational performance and achievable accuracy, the input images were rigidly aligned and resampled on a 256 × 256 × 256 grid prior to registration, with the resulting DVFs being resampled at the original image dimensions before employing them for contour propagation. The weighting parameters α and β were set empirically at 0.3 and 1.0, respectively. For an analysis of auto-contouring accuracy as a function of α and β see Figure S.1 in the supplementary material.

Both DL inference and the DIR were executed on a nVidia RTX A5000 GPU, with the registration step being implemented via the compute unified device architecture (CUDA, version 11.8) framework.^1^

### 2.3 Evaluation of the proposed auto-contouring framework

EvoDL was initially evaluated before clinical implementation (pre-implementation), providing information on its safety and expected performance, followed by implementation and evaluation during its clinical operation (post-implementation). Before clinical release, the software was made to conform to the medical device regulations via the ISO13485-certified quality management system implemented within our department.

#### 2.3.1 Pre-implementation evaluation

Before its clinical release, EvoDL was evaluated on data acquired on ten prostate cancer patients undergoing a 5×7.25 Gy MR-guided RT at the UMC Utrecht. This was the same data used in a previous study [14], where the Evolution algorithm, without contour guidance, was evaluated for contour propagation.

Contour propagation using EvoDL was performed both inter- fraction, between the planning and the daily T2-weighted MR images, as well as intra-fraction, between 2–3 position verification images, resulting in the framework being applied on a total of 105 image pairs (50 inter-fraction + 55 intra-fraction). The propagated contours provided by the framework were evaluated by a radiation oncologist (the same as in [14]) and scored using the following Likert scale: 0 - no adjustments, 1 - few minor adjustments, 2 - multiple minor/few major adjustments, 3 - multiple major adjustments. The evaluation was performed individually for the CTV, bladder, and rectum, within a 2 cm ring centered on the CTV, in line with the contour verification protocol employed in the department. In addition to the aforementioned Likert scale, it was also determined whether the contour correction and verification time was possible within 2 min.

#### 2.3.2 Post-implementation evaluation

In between November 2024 and January 2026 a total of 275 prostate cancer patients completed the prescribed 5×7.25Gy MR-guided RT treatment, with EvoDL being used as the daily auto-contouring tool. Following the initial contour proposal, the CTV, bladder, and rectum contours were verified and corrected within a 2 cm ring centered on the CTV by the RTT delivering and monitoring the treatment. During the first 3.5 months following its clinical introduction (up to March 2025), the RTTs have also recorded the required contouring time, consisting of the auto-generation of the contours plus the contour correction time, for 59 patients.

All of the resulting data was retrospectively analyzed, with the quality of the contours proposed by the new framework being quantified by comparing the initial contour proposals with their corresponding clinically-approved counterparts. This was performed both in terms of the Dice similarity coefficient (DSC) as well as the 95% Hausdorff distance (HD) [21], [22]. Moreover, the duration of the contouring step, using the previously-employed vendor-provided solution (Monaco 6.2.1, Elekta Inc., Sunnyvale, California, USA), was also available for a separate cohort of 34 patients which underwent the same treatment scheme (between September–November 2024) and was used as a point of comparison. Statistical significance of the contouring time differences between EvoDL and Monaco 6.2.1 was verified using a Mann–Whitney statistical test [23].

The variation of the contouring time across the five fractions was also investigated, with statistical significance of timing differences having been evaluated via a Kruskal–Wallis statistical test [24]. Finally, changes in contouring time during the 3.5 months following clinical implementation as well as long-term time tendencies (via a linear fit of the contour times) were also evaluated.

For all statistical tests, a *p-value* of less than 0.05 was considered statistically significant.

## 3 Results

According to the qualitative pre-implementation evaluation, for the majority of the cases EvoDL required no or minor adjustments, with only 4 (3.8%) requiring a few major adjustments for the CTV and 2 (1.9%) requiring multiple major adjustments for the bladder (Table 2). The rectum contour required adjustment for only 4 out of all 105 cases. Overall, the number of instances in which any of the contours required any major adjustments was considerably low relative to the total number of evaluations. Even so, in terms of timing, all of the adjustments were achieved within two minutes per case.

During the post-implementation evaluation, the majority of the contours underwent only minor adjustments, showcased by both the DSC and HD95 (Table 3), where 75% of the DSC values are higher or equal 0.95, with the corresponding HD95 being under 2.13 mm. The outcomes of identical analyses carried out only for the guiding contours as well as for Evolution, without contour guidance, are provided in the supplementary materials (Tables S.1 and S.2).

**Table 2:** Outcome of the pre-clinical evaluation of EvoDL, in terms of the number and level of adjustments required for the generated contours. Evaluation and reporting was performed for the CTV and the clinically-relevant OoI (i.e. bladder and rectum).

Adjustments needed	CTV	Bladder	Rectum
No adjustments needed (0)	70	91	101
Few minor adjustments needed (1)	31	12	2
Multiple minor/few major adjustments needed (2)	4	0	2
Multiple major adjustments needed (3)	0	2	0
Total	105	105	105

There were however several instances in which multiple major adjustments were required, as reported by the treating RTT. The root cause, as determined in retrospect, was either due to a failure of the DL component to provide a reliable contour for the bladder (in 10 out of 1375 fractions) or due to the registration being unable to resolve particularly large displacements between the planning and the daily MR images (in 9 out of 1375 fractions).

A typical example of EvoDL output as well as each type of the two aforementioned defects is shown in Figures S.2, S.3 and S.4 in the supplementary material.

EvoDL also significantly reduced the time required for end-to-end contouring after clinical implementation (*p-value* <0.01), compared to Monaco 6.2.1, from a median value of ≈470 s to ≈190 s (Fig. 2a). Each measurement consists of the computational latency of the software (≈90 s on average) together with the contour correction time by the RTT. From Fig. 2a, it can be observed that several outliers are also present in the timing data, corresponding to instances in multiple major adjustments were necessary (as mentioned previously).

**Table 3:** Overlap between the original contours generated by EvoDL and their clinically approved counterparts, evaluated via the: (a) Dice similarity coefficient; (b) The 95% Hausdorff distance.

(a) Dice similarity coefficient							
Structure	Min.	5th Qu.	25th Qu.	Median	75th Qu.	95th Qu.	Max
bladder_ring_	0.00	0.84	0.97	0.98	0.99	0.99	1.00
rectum_ring_	0.00	0.94	0.97	0.97	0.98	0.98	1.00
CTV	0.42	0.92	0.95	0.96	0.97	0.98	0.98

(b) Hausdorff 95% [mm]							

bladder_ring_	0.00	0.35	0.70	0.70	0.70	4.00	24.73
rectum_ring_	0.00	0.70	0.70	0.70	0.70	2.05	24.00
CTV	0.62	0.84	1.40	2.00	2.13	4.45	24.22

Considering the statistical distribution of the time required for contouring per individual fraction (Fig. 2b), no significant differences of the contouring time on the fraction number has been observed (*p-value* =0.85).

Finally, Fig. 2c showcases the contouring time over the 3.5 months following the clinical introduction of EvoDL. It can be noticed that the contouring time has remained relatively stable, with only a marginal tendency to increase over time and no evident pattern regarding the occurrence of outliers.

## 4 Discussion

The current study presented the implementation and clinical evaluation of an auto-contouring workflow for MR-guided daily-adaptive prostate radiotherapy. The developed EvoDL software combined a GPU-accelerated DIR method with contours originating from a 3D nnUnet, leveraging the complementary advantages of both deep learning segmentation and DIR. EvoDL demonstrated high accuracy, with its clinical implementation having reduced the contouring time by more than half compared to the previous vendor-provided solution.

In a similar study, Konrad et *al* have reported the outcome of a purely AI-based solution implemented at their institute [13], having achieved a contouring time reduction from 9.8 to 5.3 min, versus 7.8 min to 3.1 min for EvoDL. However, the organs-of-interest in [13] were delineated within a 3 cm ring (1 cm superior and inferior) around the prostate (versus 2 cm isotropic in our protocol), which may have an impact on the relative contouring time. Our analysis has also revealed no significant variation of the contouring time over the five fractions and only a marginal increase on the long-term (see Fig. 2). Aside the AI solution in [13] and the present study, there is currently a limited amount of literature reporting the systematic clinical performance of auto-contouring solutions for daily MR-guided prostate radiotherapy, which could make the topic of future studies. While a wide-range of research-grade algorithms were previously proposed and evaluated, variability in workflow, protocol and/or patient population makes a direct comparison in terms of their relative contour approval time particularly challenging.

The pre-implementation analysis has also shown that the majority of the contours required no or only minimal adjustments, showcasing an improvement over using the DIR component alone, as investigated in one of our previous studies [14]. Post-implementation, the DSC and HD95 between the original proposal and their clinically-approved counterparts was above 0.95 and under 2 mm, respectively, for more than 75% of the cases. This is well in line with the recommendations of task group AAPM 132, as well as other similar guidelines, for use of DIR software in image-guided adaptive radiotherapy [18], [25], [26]. Of note are however also the 19 instances in which the initial EvoDL proposal was particularly poor. This was due to: (1) The DL network severely failing to identify the contour of the bladder on the daily MR image (10 cases) and therefore having a strong influence on the resulting DVFs or (2) The registration component being unable to accurately resolve large displacements (>2.5 cm) between the planning and the daily image (9 cases). These, however, were well detected and corrected by the RTT and thus having no impact on treatment safety.

In terms of the hybrid design of EvoDL, the employed nnUnet model was selected due to its previously demonstrated performance both in terms of accuracy and inference time, for the purpose of MR-guided prostate RT [11]. Similarly, the Evolution algorithm was chosen due to its previous performance for MR-based RT guidance [14], [18], [20]. Both components work synergistically, with the DL network providing an estimate of the bladder and rectum boundaries, which are then used in conjunction with the anatomical information encoded in the data fidelity term of Evolution to generate and refine the propagated contour proposal. This aimed to address a previously-observed limitation of the Evolution algorithm which, as shown [14], had difficulties in estimating large volumetric changes occurring withing the bladder and rectum, aspect which was further emphasized by their overall lower DSC and higher HD95 values in supplementary Table S.2. Moreover, by pairing a DL-based contouring solution with a DIR method, this provides access to a set of underlying deformation vector fields, which can be re-purposed for other tasks such as inter-fraction dose accumulation and/or warping of other quantitative information.

EvoDL is also expected to be easily generalizable, as the DIR component (i.e. Evolution) has previously shown consistent accuracy for several anatomical sites and image modalities/contrasts [18], [20], [27]. The 3D nnUNet generating the guiding contours would most likely have to be re-trained if significant changes occur in terms of anatomy, modality or MR image contrast. However, given the current availability of pre-trained (foundational) models, it is not expected that requirements on training data will pose a major challenge.

In conclusion, the implemented approach has showcased a high degree of accuracy, well in line with guidelines for use of DIR technologies in image-guided adaptive radiotherapy. Additionally, the median time duration required for contouring was practically halved, compared to the previously-employed vendor-provided solution. Even so, it has to be taken into consideration that contour verification by a human operator can incur time delays of 90–120 s, even in the absence of any adjustments. In this sense, future workflows could consider the employment of “light” adapt-to-shape approaches [28], [29], which imply the correction of a particular contour only if it lies outside of a pre-defined margin, rather than the operator aiming to replicate the planning delineations onto the daily anatomy as close as possible. For the foreseeable future, our RT department will continue to use EvoDL as part of the adaptive workflow for 5×7.25 Gy MR-guided prostate RT treatments, with possible extensions to other treatment regiments, sites and modalities being currently explored and developed.

## CRediT authorship contribution statement

**Cornel Zachiu:** Writing – original draft, Visualization, Validation, Software, Methodology, Investigation, Formal analysis, Data curation, Conceptualization. **Gijsbert H. Bol:** Writing – review & editing, Validation, Software, Methodology, Data curation, Conceptualization. **Alexis N.T.J. Kotte:** Writing – review & editing, Validation, Software, Methodology, Data curation, Conceptualization. **Thomas Willigenburg:** Writing – review & editing, Validation, Investigation, Formal analysis. **Matteo Maspero:** Writing – original draft, Software, Methodology. **Mark H.F. Savenije:** Writing – review & editing, Software, Methodology, Data curation. **Johannes C.J. de Boer:** Writing – review & editing, Resources, Data curation, Conceptualization. **Jochem R.N. van der Voort van Zyp:** Writing – review & editing, Validation, Resources, Investigation, Formal analysis. **Cornelis A.T. van den Berg:** Writing – review & editing, Resources, Methodology, Conceptualization. **Bas W. Raaymakers:** Writing – review & editing, Supervision, Resources, Project administration, Methodology, Conceptualization.

## Declaration of competing interest

The authors declare the following financial interests/personal relationships which may be considered as potential competing interests: Our department has a research agreement with Elekta AB. Elekta AB had no role in the preparation, review or approval of the manuscript and the decision to submit the manuscript.

## Appendix A Supplementary data

MMC S1 Supplementary material EvoDL. MMC S1

## Footnotes

1 A research version of EvoDL is accessible at https://github.com/czachiu/deformable-registration/. A model card for the deep learning component is available from https://doi.org/10.5281/zenodo.21787146.
