# Supplementary material for: Deep learning-enhanced image registration for accelerating daily adaptive magnetic resonance imaging-guided prostate radiotherapy
Source: Phys Imaging Radiat Oncol. 2026 Aug 2;40:101052. doi: 10.1016/j.phro.2026.101052 (PMC13486320; doi:10.1016/j.phro.2026.101052)
Supplement: Supplementary file 1 [file mmc1.pdf]

Supplementary material

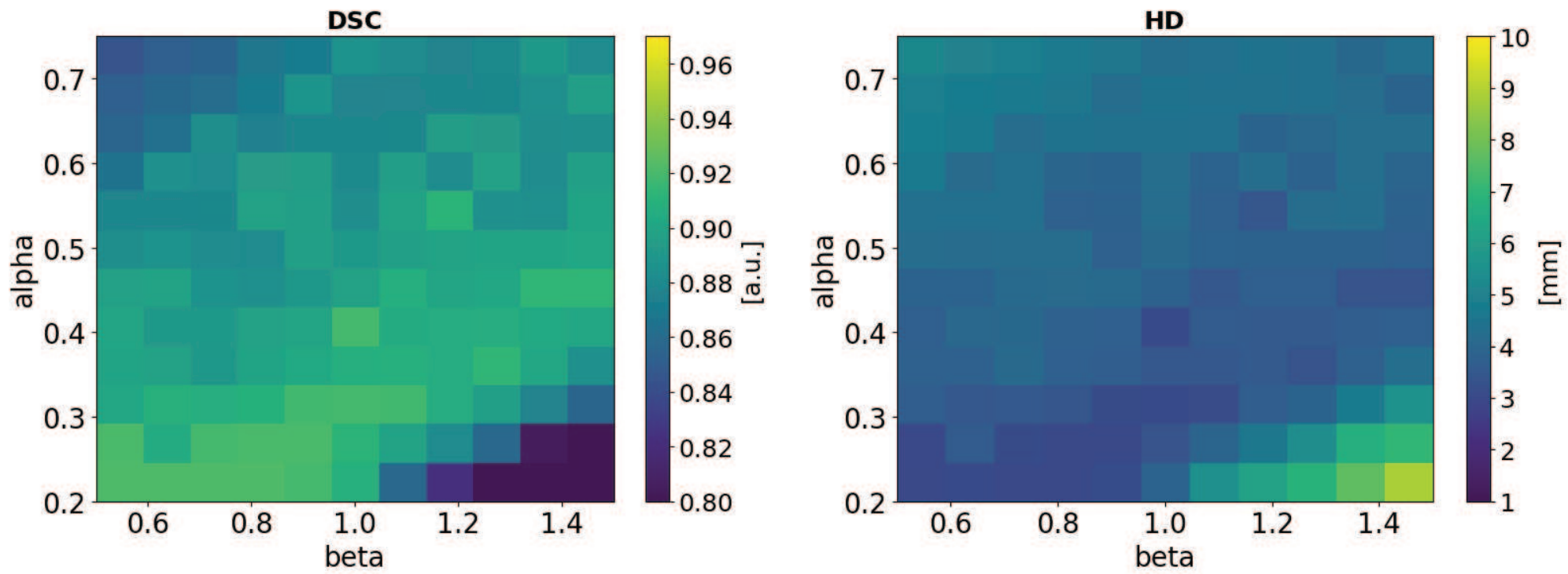

(a) (b)

Figure S.1: Dependency of the EvoDL accuracy on the values of the configuration parameters α and β from Equation 1 in the manuscript. The two heatmaps showcase the (a) Dice similarity coefficient (b) Hausdorff 95% as a function of α and β. Each value in the two heatmaps is the result of averaging the DSC and HD95 respectively, over the bladder, rectum and CTV contours from all fractions of five random patients. The configuration used in the clinical version of the tool is $\alpha = 0.3$ and $\beta = 1.0$.

Structure	Min.	5th Qu.	25th Qu.	Median	75th Qu.	95th Qu.	Max
$\text{bladder}_{\text{ring}}$	0.00	0.85	0.97	0.98	0.98	0.99	0.99
$\text{rectum}_{\text{ring}}$	0.68	0.94	0.97	0.97	0.98	0.98	0.99

(a) Dice similarity coefficient

Structure	Min.	5th Qu.	25th Qu.	Median	75th Qu.	95th Qu.	Max
$\text{bladder}_{\text{ring}}$	0.00	0.70	0.70	0.70	1.70	4.00	273.47
$\text{rectum}_{\text{ring}}$	0.62	0.70	0.70	0.70	0.99	2.06	16.88

(b) Hausdorff 95% [mm]

Table S.1: Accuracy of the bladder and rectum guiding contours generated by the nnUNet on the 275 patients included in the study: (a) Dice similarity coefficient; (b) The 95% Hausdorff distance between the deep learning-generated guiding contours and their corresponding clinically-approved counterparts.

Structure	Min.	5th Qu.	25th Qu.	Median	75th Qu.	95th Qu.	Max
$\text{bladder}_{\text{ring}}$	0.00	0.69	0.89	0.94	0.96	0.98	0.99
$\text{rectum}_{\text{ring}}$	0.00	0.71	0.87	0.92	0.94	0.96	0.98
CTV	0.00	0.85	0.93	0.95	0.96	0.97	0.98

(a) Dice similarity coefficient

Structure	Min.	5th Qu.	25th Qu.	Median	75th Qu.	95th Qu.	Max
$\text{bladder}_{\text{ring}}$	0.00	0.99	2.00	2.00	3.10	6.82	23.94
$\text{rectum}_{\text{ring}}$	0.70	1.40	2.00	2.63	4.77	10.50	25.52
CTV	0.70	1.40	2.00	2.05	2.70	6.44	30.06

(b) Hausdorff 95% [mm]

Table S.2: Accuracy of the bladder, rectum and CTV provided by the Evolution algorithm, without contour guidance, on the 275 patients included in the study: (a) Dice similarity coefficient; (b) The 95% Hausdorff distance between the generated contours and their clinically-approved counterparts.

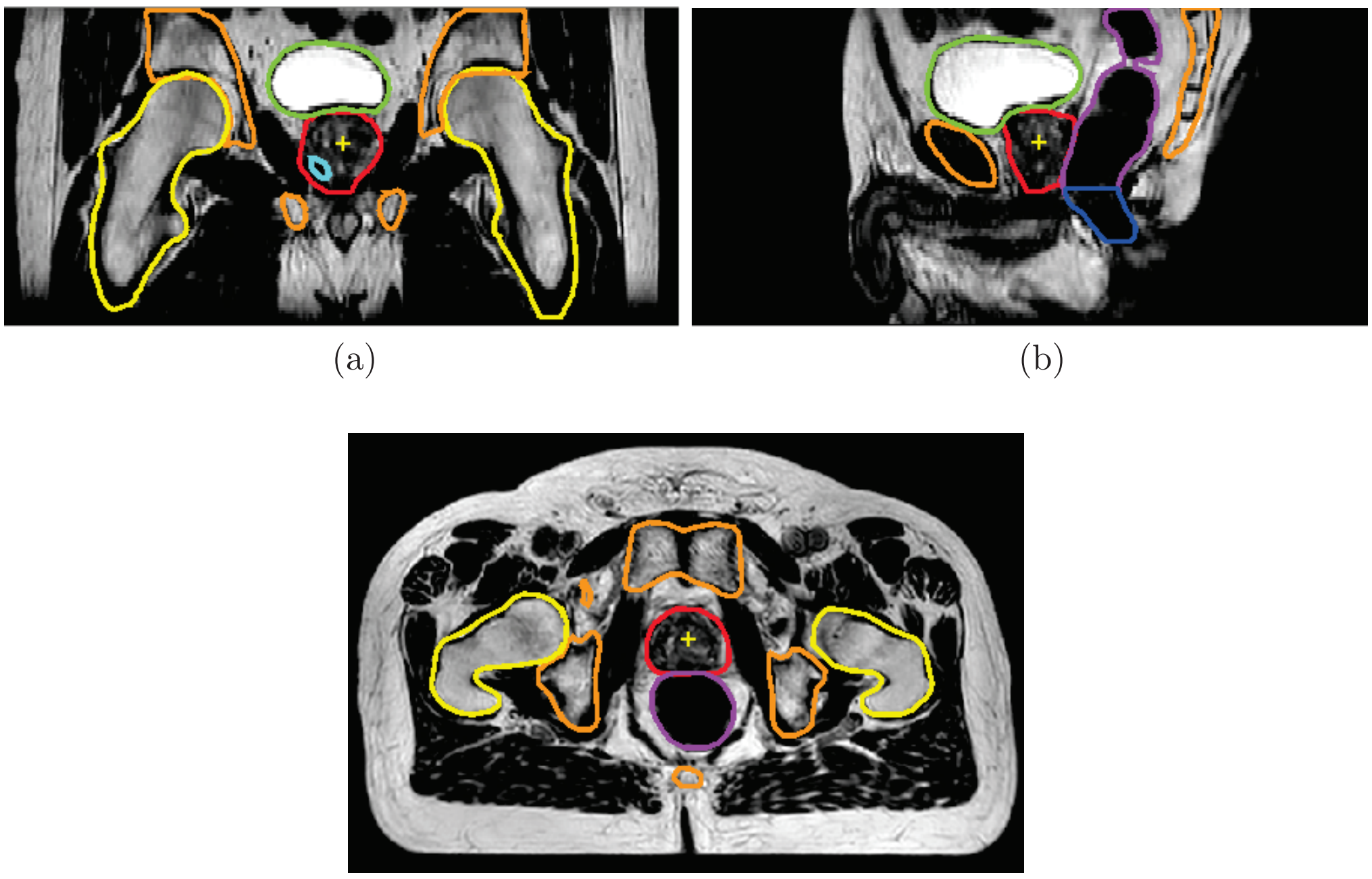

(a) (b) (c)

Figure S.2: An example of the contouring output provided by EvoDL. The figure illustrates a (a) coronal; (b) sagittal and (c) axial slice from a daily MR image, with an overlay of the propagated contours. Color coding is as follows: GTV (cyan), CTV (red), Bladder (green), Rectum (magenta), Sphincter (blue), Femurs (yellow), Pelvic and pubic bones (orange).

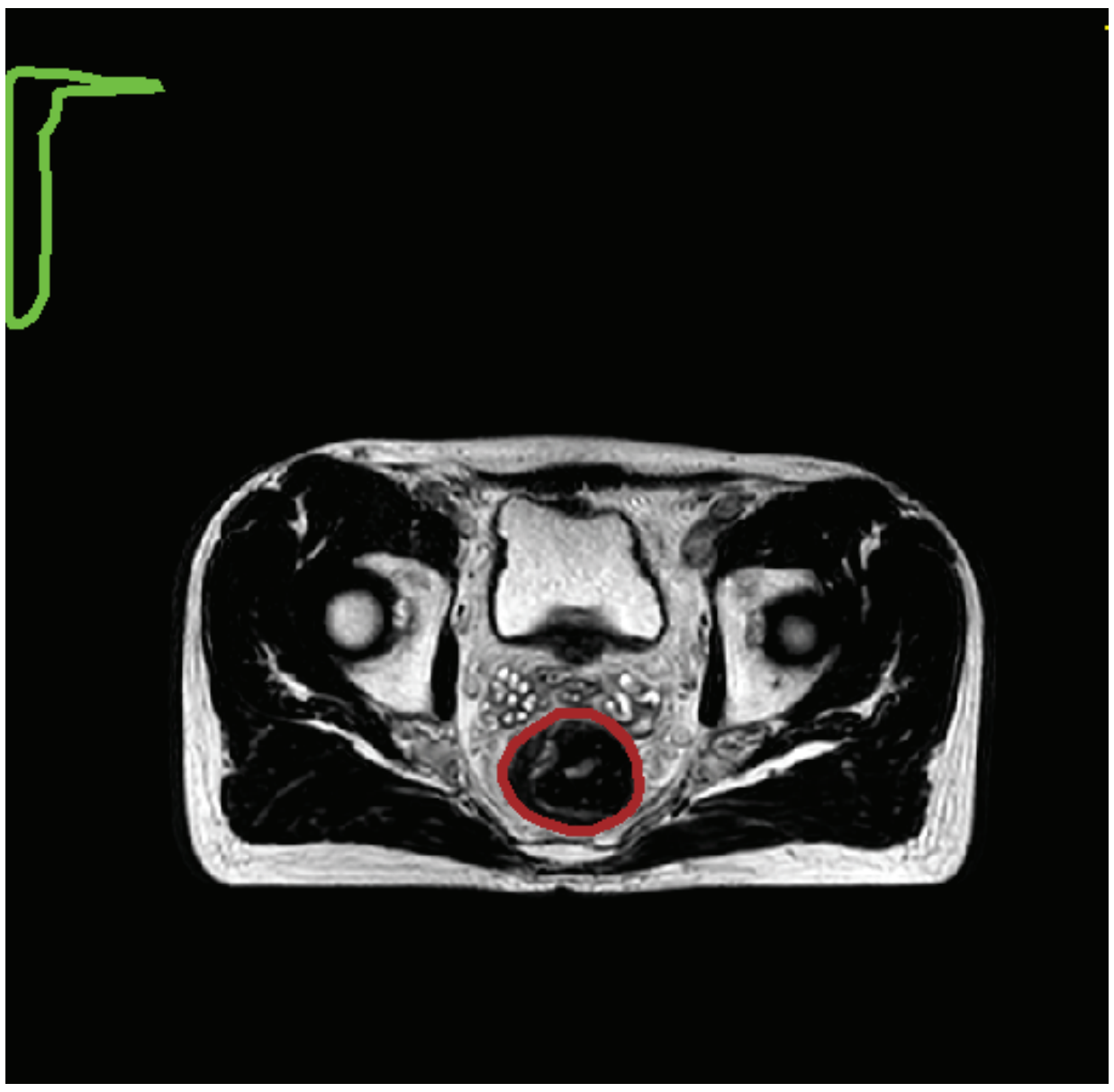

Figure S.3: Typical example of the nnUNet failing to provide a reliable guiding contour for the bladder which, on this image, has been segmented as being an area of the background (green contour in the upper left part of the figure). The same artifact was observed for all 10 instances in which the deep learning component of the software was determined to have failed.

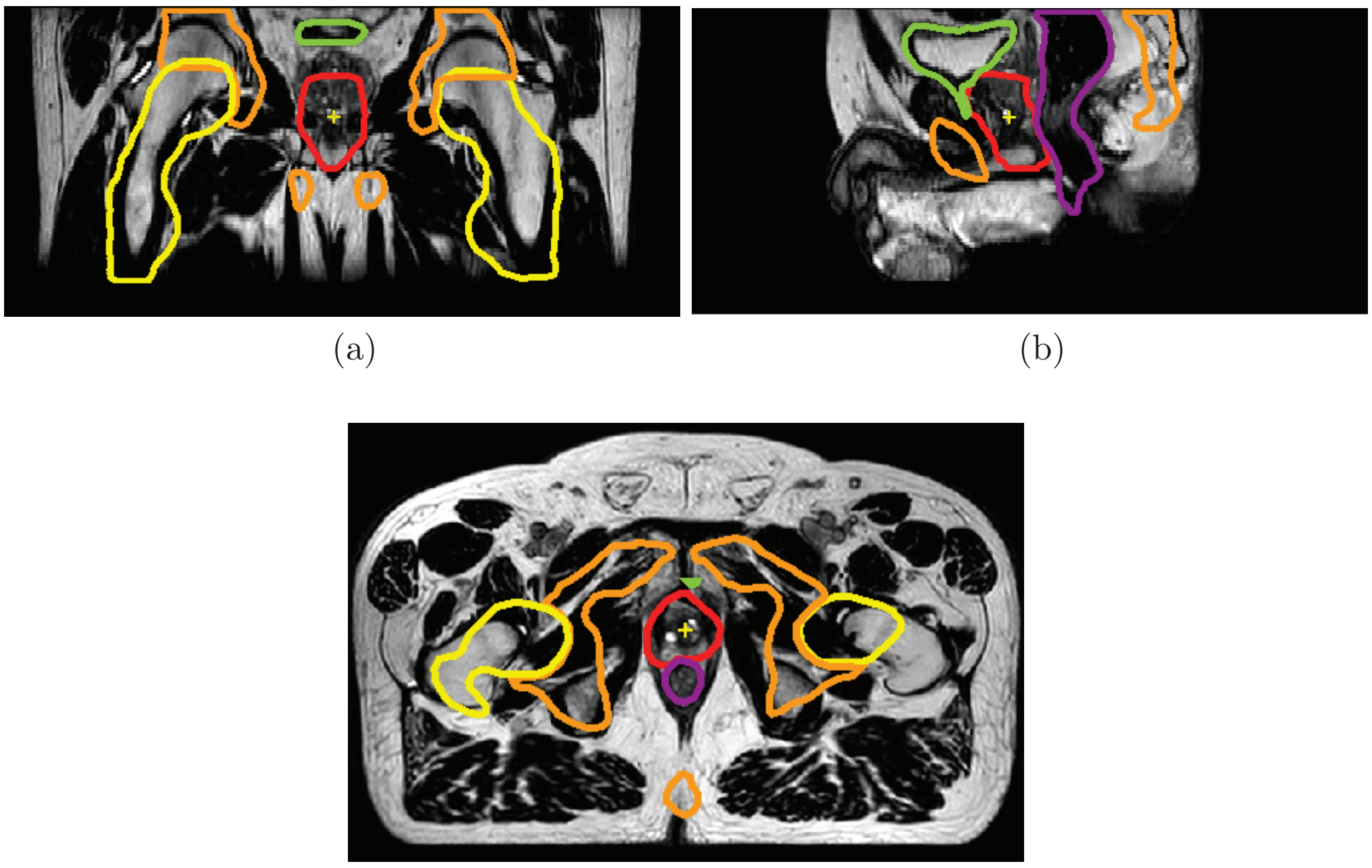

(a) (b) (c)

Figure S.4: Typical example of an instance in which the registration component of the software was determined to have failed to provide a reliable alignment between the planning and the daily image. The figure illustrates a (a) coronal; (b) sagittal and (c) axial slice from a daily MR image, with an overlay of several of the propagated contours. A large misalignment between the propagated femur and pelvic bone contours (in yellow and orange, respectively) and the underlying anatomy can be observed. The propagated CTV contour (in red) also extends into the corpus spongiosum in the caudal direction. A near-identical behavior has been observed for all 9 instances in which the registration component was determined to have failed.
